# Salivary Extracellular Vesicles: Paradigm Shift in Liquid Biopsy Diagnostics

**DOI:** 10.1002/jex2.70166

**Published:** 2026-06-30

**Authors:** Kwanele Xulu, Usri H. Ibrahim, Irene Mackraj, Carola Niesler

**Affiliations:** ^1^ Department of Human Physiology School of Laboratory Medicine and Medical Sciences University of KwaZulu‐Natal Durban South Africa; ^2^ Discipline of Biochemistry School of Agriculture and Science University of KwaZulu‐Natal Pietermaritzburg South Africa

## Abstract

Extracellular vesicles (EVs), lipid bilayer nanoparticles released by virtually all cells, serve as essential messengers for intercellular communication. Due to their involvement in several pathophysiological processes, EVs have recently gained considerable attention as potentially diagnostic and prognostic biomarkers for various illnesses. The widespread distribution of EVs across all biofluids positions them as ideal, minimally invasive biomarkers for disease progression using a liquid biopsy approach. Among biofluids, saliva is uniquely accessible and has a low soluble protein content, making its EV population a highly promising source of diagnostic biomarkers. Salivary EVs have been investigated for their promising potential for diagnosing local and systemic diseases, including cancers, autoimmune diseases and neuropsychiatric disorders. In this review, we present a synopsis of the current landscape related to salivary EVs, highlighting the unique characteristics and potential of these vesicles, the technical challenges related to their application, and their future prospects for clinical translation as powerful diagnostic tools.

## 1 Introduction

Extracellular vesicles (EVs) typically range in size from 30 to 1000 nm and are now recognised as crucial mediators of intercellular communication, transporting a diverse array of bioactive cargoes including nucleic acids, proteins, and lipids (Buzas 2023; Dorado et al. 2024; Hánělová et al. 2024). This understanding, however, is the product of decades of incremental discovery across disparate biological contexts.

The earliest observations that would retrospectively inform the EV field emerged from haematology. In 1946, Chargaff and West identified platelet‐derived particles with procoagulant activity, and two decades later Wolf (1967) described the vesicular ultrastructure of these minute particulate fractions, termed ‘platelet dust’, and confirmed their clotting activity, collectively suggesting that these membrane particles possess biological function. Extending this idea beyond blood, Anderson (1969) identified membrane‐bound ‘matrix vesicles’ derived from chondrocytes within the cartilage matrix of the tibial epiphyseal plate and proposed a role for these structures in tissue mineralisation, establishing that vesicle‐mediated biological activity was not confined to the circulatory system. Further evidence for vesicle secretion came from Ronquist et al. (1978), who described round to oval, membrane‐bound vesicles, later termed prostasomes, in prostatic and seminal fluids; Brody et al. (1983) subsequently demonstrated their prostatic cellular origin, lending these structures both structural and biological credibility.

A pivotal conceptual shift came in the early 1980s when the endosomal origin of a subset of EVs began to be recognised. Nunez et al. (1974) had earlier noted multivesicular bodies (MVBs) in the secretory cells of bat thyroid gland and hinted at the secretion of intraluminal vesicles through MVB fusion with the apical plasma membrane, anticipating what would become a defining biogenesis pathway. Building on this, Pan and Johnstone (1983) demonstrated that reticulocytes selectively externalize transferrin receptors via small membrane vesicles during maturation, a finding elaborated upon by Harding et al. (1983), who further characterised the endosomal pathway underlying this process. Johnstone et al. (1987) subsequently coined the term ‘exosome’ specifically for these vesicles of endosomal origin, providing the field with its first formal nomenclature for what is now recognised as a distinct EV subtype. In parallel, Trams et al. (1981) had introduced the term ‘exosome’ independently to describe microvesicles expressing ecto‐5`‐nucleotidase in cell culture superfusates. This was a terminological parallel that reflects the concurrent convergent nature of early EV discovery across different experimental systems.

These studies collectively laid the groundwork for the consolidation of EV biology in the 1990s. Raposo et al. (1996) provided a particularly influential contribution by demonstrating that EVs participate in antigen presentation, repositioning them from merely cellular debris to functional immune mediators. In the early 2000s, the recognition of EVs as active participants in both physiological and pathological processes stimulated a broader interest in the field, culminating in the establishment of the International Society for Extracellular Vesicles (ISEV) in 2012. The society subsequently published its first Minimal Information for Studies of Extracellular Vesicles (MISEV) guidelines in 2014, with revisions in 2018 and 2023, providing a dynamic, community‐driven framework for standardising nomenclature, isolation methods, and reporting requirements to improve the rigour and reproducibility of EV research (Lötvall et al. 2014; Théry et al. 2018; Welsh et al. 2024).

The functional versatility of EVs as mediators of intercellular communication is associated with the diversity of their biogenesis pathways and the range of mechanisms through which they interact with recipient cells. EVs are understood to arise through multiple distinct mechanisms, each producing populations with a characteristic size range, surface composition, and cargo profile. The most well‐characterised pathway produces exosomes, which originate through inward invagination of the endosomal membrane to form intraluminal vesicles within MVBs; these are released into the extracellular space upon fusion of the MVB with the plasma membrane (Colombo et al. 2013; Ostrowski et al. 2010). A parallel, well‐established route generates ectosomes (microvesicles) through the direct outward budding and scission of the plasma membrane (Mathieu et al. 2021). Apoptotic bodies represent a third EV population released as a consequence of programmed cell death, and they are typically larger than other EV subtypes (Santavanond et al. 2021). Beyond these established categories, the field has increasingly recognized a broader landscape of extracellular particles. Secretory autophagosomes (autophagic extracellular vesicles) are proposed to originate from amphisomes formed by the fusion of autophagosomes with MVBs, positioning autophagy as a contributor to EV output (Mao et al. 2025). Migrasomes, vesicular structures that form in the retraction fibres at the rear of migrating cells, have also been reported (Ma et al. 2015; Van Niel et al. 2022), though their classification within the EV taxonomy remains actively contested (Gudbergsson and Etzerodt 2024; Zhang et al. 2024). Further extending this landscape, exomeres and supermeres have been identified as non‐vesicular nanoparticles whose biological functions remain to be fully characterized (Yu et al. 2025; Zhang et al. 2019). Collectively, this expanding taxonomy reflects a field moving away from a simple two‐pathway model toward multiple pathways which cells utilize to package and release extracellular material.

Upon release, EVs must interact with and/or be taken up by recipient cells, a process that is equally heterogeneous and context‐dependent. Several mechanisms have been proposed, including clathrin‐mediated endocytosis, caveolin‐dependent endocytosis, micropinocytosis, lipid raft‐mediated internalisation, and phagocytosis (Feng et al. 2010; Mulcahy et al. 2014). It is now also recognised that receptor‐ligand interactions at the EV surface can facilitate cellular uptake (Hallal et al. 2022; Théry et al. 2002; Utsugi‐Kobukai et al. 2003). The relative contribution of each pathway appears to vary with cell type, EV surface composition, and the physiological context of the interaction; no single mechanism has been established as universally dominant. This context‐dependence is biologically meaningful as it suggests that uptake selectivity may itself be a regulated feature of EV‐mediated signalling rather than a passive consequence of vesicle encounter with a target cell. Together, the diversity of biogenesis and uptake mechanisms positions EVs as an intriguing and adaptable system for intercellular communication, one whose complexity is still being defined.

Unlike many analytes present in biofluids, EV cargo is shielded from enzymatic degradation by the vesicle's lipid bilayer, while retaining the capacity to induce phenotypic and genetic changes in recipient cells. This protective encapsulation, combined with the ability of EVs to mirror the molecular state of their cell of origin, underpins their growing appeal as biomarkers across a wide range of conditions (Hoshino et al. 2020; Thietart and Rautou 2020; Thompson et al. 2016; Xu et al. 2020). Circulating through the lymphatic and circulatory systems, EVs gain access to multiple biofluids through various mechanisms (Iannotta et al. 2024) and have been reported to cross the blood‐brain‐barrier (Banks et al. 2020; Ramos‐Zaldívar et al. 2022), extending their reach to compartments traditionally considered inaccessible to other circulating factors.

Among the biofluids in which EVs have been studied, saliva occupies a distinctive position. It is readily accessible through non‐invasive collection methods, removing some of the procedural barriers associated with venepuncture or lumbar puncture, and can be sampled repeatedly over time to monitor disease progression. Whole saliva is a compositionally rich mixture, comprised of secretions from the major and minor salivary glands (Pedersen et al. 2018; Uchida and Ovitt 2022), gingival crevicular fluid (Majeed et al. 2023; Taylor and Preshaw 2016), oral microorganisms and their products (Dong et al. 2022; Marcotte and Lavoie 1998), and cellular debris (Ekström et al. 2017), making it a potentially information‐dense source of disease biomarkers. Relative to blood, saliva is also less complex, with no post‐collection EV pool confounding by activated platelet‐derived EVs (Małys et al. 2023). Despite these practical and biological advantages, salivary EVs remain considerably underexplored compared to their counterparts in blood or urine. As a result, the field is still in the process of establishing the analytical and clinical frameworks necessary to translate salivary EV findings into diagnostically actionable tools.

Cancer represents one of the most actively investigated areas in this context, given that late‐stage diagnosis remains a key driver of poor outcomes. The field has therefore long sought minimally invasive strategies for early detection. In this regard, salivary EVs have attracted interest as a potential window into tumour biology and progression. Ono et al. (2020) reported that EVs derived from metastatic oral cancer cells, enriched with molecular chaperons including CD37, HSP90α, and HSP90β, were associated with macrophage M2 polarisation, epithelial‐mesenchymal transition (EMT), and tumour cell migration and invasion in experimental models. These intriguing findings represent one proposed mechanism in a specific model system, and such findings require replication in independent systems and clinical correlation before their translational significance can be assessed.

This extends to the wider candidate biomarker literature, where promising signals are frequently identified in small, exploratory cohorts. Sun et al. (2016), for instance, used proteomic profiling of saliva from lung cancer patients to identify several candidate proteins for lung cancer‐associated biomarkers; however, the study's sample size of nine patients limits the ability to draw conclusions regarding their biomarker robustness. In a related line of investigation, Sun et al. (2018) proposed that salivary EVs carry proteins originating from distant organ cells, including BPI fold‐containing family A member 1 (BPIFA1), Cornulin (CRNN), Mucin 5B (MUC5B), and IQ motif‐containing GTPase‐activating protein (IQGAP1), as potential and could therefore potentially serve as non‐invasive indicators of lung cancer, a finding that raises broader questions about mechanisms by which distally‐derived signals reach oral compartments, and whether saliva may serve as a systemic, rather than merely local, diagnostic medium.

Beyond oncology, salivary EVs have been investigated in neurodegenerative and neuropsychiatric research (Rastogi et al. 2023), immunology (Aqrawi et al. 2017; Yu et al. 2018), and endocrinology (Byun et al. 2022; Röhrborn et al. 2024). Müller Bark et al. (2023) identified four candidate proteins (aldolase A, 14‐3‐3 protein ε, enoyl CoA hydratase 1, transmembrane protease serine 11B) in salivary EVs from glioblastoma patients associated with poor prognosis, with aldolase A showing high sensitivity and specificity in a glioma cohort. In Parkinson's disease research, Cao et al. (2019) reported elevated oligomeric α‐synuclein in salivary EVs from affected patients and proposed this as a potential screening tool for pre‐symptomatic detection, while Rastogi et al. (2023) demonstrated a cost‐effective fluorescent‐dye‐based quantification approach for identifying prodromal cases. Considered together, these studies illustrate the breadth of conditions for which salivary EV‐driven signals have been investigated as diagnostic or prognostic candidates. Given the infancy of the salivary EV field, a common limitation of these studies emerges: small, often single‐centre cohorts; cross‐sectional designs that preclude longitudinal monitoring; and a lack of independent validation. Furthermore, sensitivity and specificity figures, when reported, are frequently derived from the same datasets used for discovery, which risks overfitting.

While fundamental research still holds great significance, the field also requires a deliberate shift toward translational development, specifically prospective multicentre studies in clinically well‐defined populations, standardization of isolation and characterization protocols, and pre‐specified analytical validity thresholds before clinical utility can be meaningfully claimed. Considering the increasing interest in EVs in different biofluids over the past decades, this review seeks to demonstrate the significance of *salivary EVs* in the context of neuropsychiatric and neurodegenerative disorders, cancers, Sjögren syndrome, and inflammatory bowel syndrome for disease detection, prognosis, and treatment.

## 2 Salivary Extracellular Vesicles Characteristics

Whether salivary EVs are fundamentally distinct from EVs in other biofluids, or whether they represent a conserved vesicular scaffold variably loaded with source‐specific cargo, is a question the field is yet to answer conclusively. Nonetheless, existing evidence seems to favour the latter, that salivary EVs are built on the same structural foundations as EVs in other biofluids, and their distinguishing characteristics, where they exist, reflect the unique physiological and immunological identity of the oral cavity rather than a novel vesicle class.

Structurally, salivary EVs conform closely to EVs from other biofluids. Transmission electron microscope (TEM) images of salivary EVs reveal cup‐shaped, round particles (Sun et al. 2017; Wang et al. 2020; Zlotogorski‐Hurvitz et al. 2015), a morphology consistent with EVs from plasma, urine, and cerebrospinal fluid (Cho et al. 2020; Lee et al. 2020; Lobb et al. 2015). Atomic force microscopy 3‐D imaging of native EV structure corroborates these findings, placing salivary EVs diameter at 40–65 nm (Iwai et al. 2016; Palanisamy et al. 2010), well within the 30–150 nm range reported across biofluids (Ansari et al. 2024; Sokolova et al. 2011; van der Pol et al. 2014). Canonical EV surface markers, including CD63, CD81, and flotillin‐1, are similarly shared with EVs from other sources (Reseco et al. 2024). None of this data suggests a distinct salivary EV structural profile.

The more diagnostic question concerns molecular cargo. Ogawa et al. (2008) first characterized salivary EVs by electron microscopy, identifying 30–130 nm vesicles varying in DPP IV, galectin‐3, IgA, pIgR, and actin (Table 1). Since whole saliva is a composite secretion, the cargo is indirectly linked to oral cavity cells. For instance, salivary gland ductal epithelial cells express both galectin‐3 (Xu et al. 2000) and DPP IV (Sahara and Suzuki 1984), while pIgR is expressed by mucosal epithelial cells (Kaetzel 2005). These attributions are plausible but remain provisional in the absence of cell‐type‐specific EV isolation or lineage tracing, and their validation in larger studies is still needed.

**TABLE 1:** Summary of the published studies that reported salivary EV characteristics.

Cargo	EVs isolation method	Analysis technique	Major findings	Reference
Proteins	‐ Ultrafiltration (Column gel‐filtration)	‐ SDS‐PAGE ‐ Western blot	Saliva contains small EVs with protein cargo such as dipeptidyl peptidase IV, galectin‐3 and immunoglobulin A.	(Ogawa et al. 2008)
	‐ Ultracentrifugation	‐ Multidimensional protein identification mass spectrometry	Proteomic analysis of salivary small EVs (exosomes) revealed that 491 proteins were detected in the exosome fraction, with 226 proteins not detected in the original saliva.	(Gonzalez‐Begne et al. 2009)
	‐ Ultrafiltration (Sephacryl S‐500 gel‐filtration)	‐ In‐gel digestion Mass Spectrometry ‐ LC‐MS/MS ‐ Shotgun proteomic analysis	Identification of two distinct populations of salivary EVs, with distinct proteomic profiles, and different physical and functional properties (EV‐1 and EV‐II)	(Ogawa et al. 2011)
	‐ Filtration ‐ Centrifugation	‐ In‐gel digestion ‐ LC–MS/MS	Identification of salivary microvesicles with a unique size and protein profile.	(Xiao and Wong 2012)
Nucleic acids	‐ Ultrafiltration (Sephacryl S‐500 gel‐filtration)	‐ NGS	Salivary EV RNA content includes miRNAs, piwi‐interacting RNAs, small nucleolar RNAs, and 47 candidate novel miRNAs	(Ogawa et al. 2013)
	‐ Ultracentrifugation	‐ RT‐qPCR	Salivary small EVs (Exosomes) contain the majority of miRNAs detected in saliva.	(Gallo et al. 2012)
	‐ Ultrafiltration (Sephacryl S‐500 gel‐filtration)	‐ NGS	Salivary EVs contain an extensive repertoire of long non‐coding RNA and protein‐coding RNAs	(Ogawa et al. 2016)
Surface Proteins	‐ Precipitation ‐ Size exclusion Chromatography	‐ Western blot	High expression of CD63 with moderate expression of CD81 and flotillin‐1	(Reseco et al. 2024)
	‐ Ultrafiltration (Sephacryl S‐500 gel‐filtration) ‐ Ultracentrifugation ‐ Immunoprecipitation	‐ Nano LC–MS/MS ‐ ELISA	Unique protein signature for salivary small EVs subpopulations: EV‐I (APN/MUC1‐rich EVs, medium/large EVs) and EV‐II (DPP IV/CD9‐rich EVs, small EVs).	(Ogawa et al. 2024)

*Note*: ELISA: enzyme‐linked immunosorbent assay; LC–MS/MS: liquid chromatography–tandem mass spectrometry; NGS: next‐generation sequencing; RT‐qPCR: real‐time quantitative polymerase chain reaction; SDS‐PAGE: sodium dodecyl sulfate‐polyacrylamide gel electrophoresis.

Gonzalez‐Begne et al. (2009) subsequently catalogued 491 proteins in parotid gland‐derived salivary EVs, 29% of which overlapped with parotid ductal saliva (Table 1). The concurrent identification of ALIX, VPS4A, heat shock proteins, ANXA4, and Rab proteins reflects the conserved MVB‐mediated biogenesis machinery shared across all cell types, while the detection of AQP5 and cytokeratins hints at a tissue‐specific layer. The fact that 72 of these proteins overlap with urinary EV proteins further underscores the cross‐biofluid conservation of the EV proteome. These observations are given a sharper quantitative definition by Sun et al. (2017), who systematically compared salivary and serum exosome proteomes by label‐free quantification, identifying 319 proteins in salivary versus 994 in serum exosomes, with approximately 80% of the salivary exosomal proteome shared with serum exosomes. This is crucial to the question of fundamental distinctiveness of salivary EVs, as these findings suggest that the salivary EV proteome is not unique to saliva, and any claims of the contrary must be built on the remaining minority fraction and on demonstrating that fraction to be functionally significant. Xiao and Wong (2012) reported 63 proteins in salivary microvesicles from the annexin, keratin, actin, immunoglobulin, and S100 families (Table 1), with ANXA1–6 detected as components consistent with their established roles in MV biogenesis rather than divergence.

**FIGURE 1:**
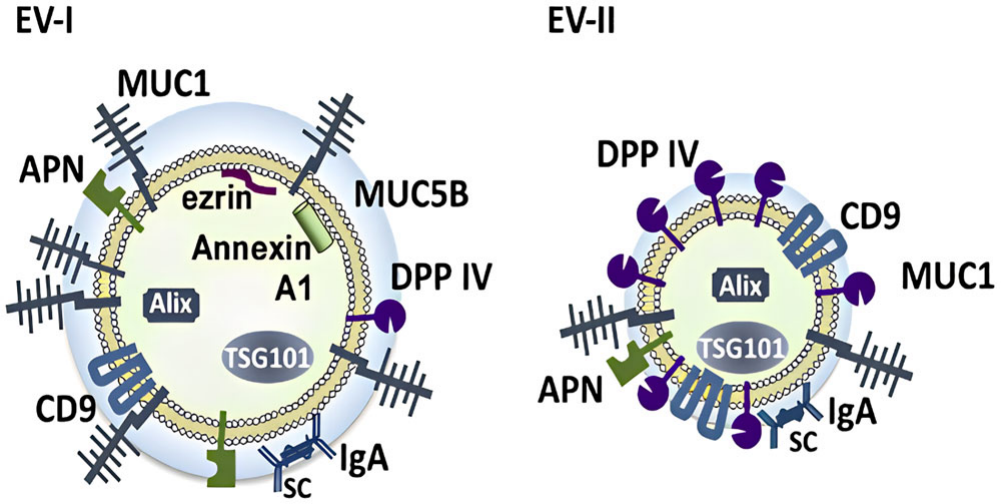
Schematic model of the two populations of extracellular vesicles (EV‐I and EV‐II) present in human whole saliva. The two subtypes differ in both size and protein composition. EV‐I is the larger EV (263 ± 30 nm) population that is enriched in aminopeptidase N (APN) and mucin 1 (MUC1). In contrast, EV‐II is the smaller (36.5 ± 13 nm) exosome‐like population that is enriched in dipeptidyl peptidase IV (DPP IV) and the tetraspanin CD9. Membrane‐associated proteins depicted include MUC1, MUC5B, APN, DPP IV, CD9, immunoglobulin A (IgA) and its secretory component (SC). Luminal proteins depicted include the canonical EV markers Alix (programmed cell death 6‐interacting protein) and TSG101 (tumour susceptibility gene 101), with ezrin and annexin A1 additionally present in EV‐I. Reproduced from Ogawa et al. (2024) under the terms of the Creative Commons Attribution license.

A more compelling case for biological source‐distinctiveness begins to emerge when protein composition of salivary EV subpopulations is examined in the context of oral mucosal immunology. Ogawa et al. (2011) identified two EV subpopulations, EV‐I and EV‐II, sharing 68 proteins including ALIX, TSG101, CD63, Hsp70, IgA, and pIgR, but differing in size and cargo. EV‐I had a diameter of 30–250 nm and was enriched in mucins (MUC5B), ezrin, moesin, radixin, α‐enolase, GNB1, annexins and Rab GDP dissociation inhibitor β, while EV‐II (20–80 nm) carried higher levels of carbonic anhydrase 6, cystatin family proteins, DPP IV, IgG Fc‐binding protein, and galectin (Table 1). These findings were extended and confirmed in a subsequent study (Ogawa et al. 2024), which re‐characterized EV‐I as aminopeptidase N/mucin 1 (APN/MUC1)‐rich medium‐to‐large vesicles and EV‐II as DPP IV/CD9‐rich small vesicles (Figure 1). The consistent identification of IgA and pIgR across both populations in two independent studies warrants attention, as these are not simply shared markers with other biofluids but markers of mucosal immune system. Secretory immunoglobulin A (SIgA) is the dominant immunoglobulin of secretory mucosae and is essentially absent from plasma, where IgG predominates; the parotid IgA‐to‐IgG concentration ratio is approximately 500‐fold higher than in serum, a direct consequence of the pIgR‐mediated transcytosis machinery that selectively concentrates dimeric IgA at mucosal surfaces (Brandtzaeg 2013). It is not arbitrary that both salivary EV subpopulations are enriched in IgA and pIgR; instead, it likely reflects the immunological identity of the cells from which these vesicles are derived. Whether this constitutes a fundamentally different EV biology or simply source‐appropriate cargo selection within a conserved biostructural framework, is a distinction the field has not yet resolved. The functional data from Ogawa et al. (2024) add further context: DPP IV is enzymatically active within salivary EVs, cleaving bioactive substrates, including C‐X‐C motif chemokine ligand 11 (CXCL11) and CXCL12 (Ogawa et al. 2011), kallidin, and substance P (Ogawa et al. 2024). Furthermore, DPP IV/CD9‐rich vesicles were shown to bind Middle East Respiratory Syndrome coronavirus (MERS‐CoV) through DPP IV and IgA, consistent with a role in mucosal surveillance and possibly antigen presentation (Ogawa et al. 2024). Whether this binding represents a programmed biological function of salivary EVs or an incidental consequence of the DPP IV‐rich mucosal environment cannot be determined from the current data. These functional observations, however, are biologically plausible and warrant further investigation as they are a starting point for mechanistic investigations rather than evidence of established function.

Salivary EVs also carry RNA cargo whose relationship to that of EVs from other biofluids remains poorly characterized. Using NGS‐based small RNA profiling from a single healthy donor, Ogawa et al. (2013) identified miRNAs, piRNAs, and snoRNAs in EV‐I, EV‐II, and whole saliva (Table 1), with miRNAs comprising 33%, 51%, and 58% of the total mapped reads, respectively. Among the 40 most abundant miRNAs per compartment, hsa‐mir‐182 was uniquely detected in EV‐I, hsa‐mir‐10b, hsa‐mir‐181a‐1, hsa‐mir‐181a‐2, and hsa‐mir‐191 in EV‐II, and hsa‐mir‐92a‐2 exclusively in whole saliva. These findings appear to contrast with those of Gallo et al. (2012), who demonstrated that salivary miRNAs are predominantly concentrated in EVs rather than EV‐depleted supernatants. However, this disparity most plausibly reflects the differences in analytical scope, as Ogawa et al. (2013); Ogawa et al. (2016) applied comprehensive total small RNA profiling across all fractions, while Gallo et al. (2012) used the sequence‐specific TaqMan assay with a predefined and limited target set. The critical constraint on interpreting this RNA data is not only due to methodological choice alone, but also due to the single‐donor design underpinning Ogawa's RNA studies, where in the absence of biological replicates, statistical inference is impossible. Additionally, identified miRNAs were not validated in independent samples and none were linked to specific source cells, target cells, or functional outcomes. The distinction between EV‐I and EV‐II RNA signatures and whether it is reproducible remains formally unconfirmed.

A further complication specific to saliva is the contribution of the oral microbiome. This necessitates rigorous analytical frameworks in salivary EV research to accurately distinguish potent microbially‐derived EVs from host cell‐derived EVs. Tong et al. (2023) reported abundant microbiome‐derived RNA across salivary EV fractions, with higher representation in microvesicles and enrichment of *Firmicutes and Bacteroidetes* sequences, extending earlier observations by Ogawa et al. (2016). In another study, Fan et al. (2023) demonstrated that *Porphyromonas gingivalis* outer membrane vesicles (OMVs) can hijack host cell machinery via microRNA‐sized small RNAs (msRNAs), specifically sRNA45033, which targets the host's CBX5 to trigger pro‐apoptotic epigenetic remodelling of the *p53* gene. Complementing this, Uemura et al. (2022) identified that *P. gingivalis* OMVs further drive chronic inflammation by delivering pathogen‐derived DNA into the host cytosol, where it activates the STING pathway. This DNA‐mediated sensing, alongside MAPK and NF‐κB signalling, significantly induces the production of pro‐inflammatory cytokines and exacerbates periodontal tissue destruction. These reports collectively implicate EVs from foreign species present in the saliva in the pathogenesis of periodontitis and suggest a role of oral microbiome‐derived EVs in oral cavity homeostasis, thus warranting further attention and discriminatory ability for EV biomarker discovery studies.

Resolving such high‐impact cross‐kingdom regulatory signals from the dense background noise of non‐sterile biofluid such as saliva remains a formidable challenge. To ascertain if microbial RNA detected in total RNA studies reflects contamination from bacterial OMVs or pathogen‐associated RNA incorporated into host EVs, principled bioinformatic workflows must be implemented. To this end, Ascensión et al. (2025) proposed a robust pipeline involving sequential host mapping, first against GRCh38 and subsequently against the CHM13 telomere‐to‐telomere assembly, to effectively purge host‐derived reads. By utilizing a consensus of multiple profilers and applying knee‐point detection algorithms (i.e., taxonomic threshold) to rank‐ordered read counts, this framework suppresses low‐abundance false positives arising from sequencing artifacts or reagent contamination. This synergistic approach, which integrates functional evidence of pathogenic DNA and RNA signalling with rigorous computational filtering, is indispensable if salivary EVs are to be successfully translated from exploratory findings into diagnostically actionable liquid biopsy factors. Such approaches would address the ambiguity between human‐derived EV cargo and bacterial OMVs, making the comparison of salivary EV RNA profiles across the studies or against EVs from sterile systemic biofluids less challenging. Beyond computational filtering, physical enrichment via immune‐affinity capture also offers a strategic avenue for enhancing the signal‐to‐noise ratio by isolating human‐specific EVs. Nevertheless, this approach introduces a significant selection bias as it restricts the analysis to specific subpopulations and potentially overlooks biologically relevant vesicles that may underexpress the chosen surface markers.

Overall, the presented evidence does not support the conclusion that salivary EVs are fundamentally different from EVs in other biofluids. The available data are more consistent with a model in which salivary EVs share the same biogenesis machinery and structural markers as EVs across the body, while carrying a subset of source‐distinctive cargo, particularly the mucosal immune proteins IgA and pIgR, mucins, and metabolically active surface enzymes that reflect the oral cavity's unique cellular and immunological environment. Whether this cargo signature is sufficient to constitute meaningful biological distinctiveness and whether it has functional consequences in recipient cells are questions the existing literature cannot answer, primarily because the studies to date have been small, largely descriptive, and not designed for cross‐biofluid comparison. What is therefore required is systematic multicentre studies that not only investigate saliva‐derived EVs, but also cross‐biofluid comparisons under standardized conditions with harmonised isolation methods and rigorous discrimination of bacterial membrane vesicles, and functional assays linking defined cargo to defined recipient cell responses.

## 3 Considerations for Salivary EVs Pre‐processing and Isolation Techniques

**FIGURE 2:**
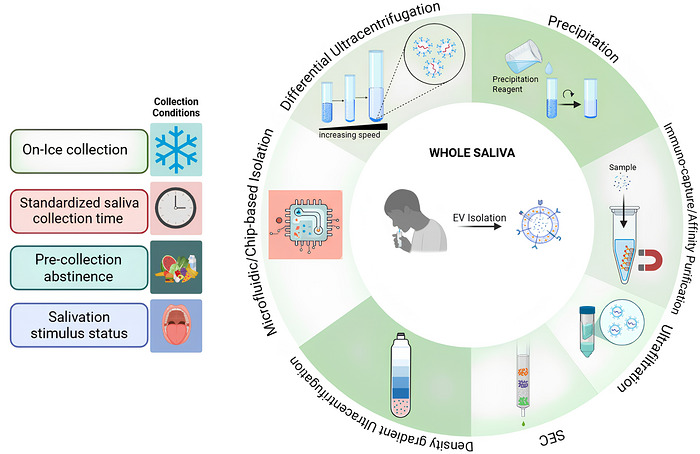
Overview of EV isolation techniques and key pre‐analytical considerations for whole saliva collection. The schematic depicts the workflow from whole saliva to isolated EVs, along with principal pre‐analytical variables that influence sample quality. Seven isolation strategies are represented: differential ultracentrifugation, polymer‐based precipitation, immune‐capture/affinity purification, ultrafiltration, size‐exclusion chromatography (SEC), density gradient ultracentrifugation, and microfluidic/chip‐based isolation. Each approach operates on distinct physicochemical principles, including size, buoyant density, precipitation, sedimentation, or affinity, resulting in variations in EV yield, purity and subtype recovery. The collection conditions panel (left) highlights four pre‐analytical factors affecting EV recovery and downstream reproducibility: on‐ice collection, standardized collection time, pre‐collection abstinence, and salivation stimulus status (stimulated vs unstimulated). The choice of isolation method should align with the study's objectives, downstream analyses, and the required balance between recovery efficiency and sample specificity. Created in BioRender.

The absence of a standardized protocol for EV isolation presents a significant challenge to their validation as biomarkers, necessitating a critical evaluation of current methodologies based on yield, purity, and feasibility. Density‐based approaches, specifically differential centrifugation (DC), remain the “gold standard” due to cost‐effectiveness and reproducibility (Figure 2, Yakubovich et al. 2022), yet this technique is hampered by labour intensity, vesicle aggregation, time, and lipoprotein co‐isolation (Sidhom et al. 2020). While density gradient centrifugation (DGC) enhances purity through isodensity fractionation (Figure 2, Brakke 1951), it suffers from low yields and potential contamination by particles of similar density (Konoshenko et al. 2018; Yu et al. 2018). Alternatively, size‐based separation strategies such as tangential flow filtration (TFF) and size‐exclusion chromatography (SEC) (Figure 2) offer advantages in structural preservation and scalability; however, TFF risks co‐filtration of contaminants (Shirejini and Inci 2022) and low yield due to loss of EVs through clogging of the filter pores (Peterson et al. 2015), while SEC often results in sample dilution and low yield (Yakubovich et al. 2022). For ease of use, polymer‐based precipitation and membrane affinity columns allow for rapid processing of low‐volume samples (Doyle and Wang 2019), though these methods frequently compromise isolation purity through the co‐precipitation of non‐vesicular proteins (Chen et al. 2022; Li et al. 2022). Conversely, immunoaffinity techniques achieve superior specificity by targeting distinct surface antigens (Sidhom et al. 2020), but their application is constrained by high costs and extended incubation requirements and enriches a specific subpopulation of EVs (Chen et al. 2022).

Similarly, different methodological approaches for the isolation of salivary EVs leverage the range of biophysical characteristics (e.g., size, density, charge, and surface composition) (Figure 2). The choice of isolation method presents a significant challenge, as techniques demonstrate a clear trade‐off between EV yield and purity. For instance, the application of density gradient methods for salivary EV isolation is reported to have high purity (Jangholi et al. 2023), while other studies have found SEC to offer the highest purity (Reseco et al. 2024), albeit at the expense of a significantly lower yield, a limitation of the SEC noted by others when working with saliva (Jangholi et al. 2023; Tengler et al. 2024). Conversely, precipitation‐based protocols may offer high salivary EV and protein recovery (Itzel et al. 2025), but this is often attributed to the co‐precipitation of non‐vesicular proteins, resulting in lower purity (Boulestreau et al. 2024; Li et al. 2021).

It is therefore critical to standardize collection protocols in order to mitigate the variability introduced by the circadian rhythms, as this influences the saliva flow rate (Papagerakis et al. 2014), saliva composition (Diab et al. 2024; Wada et al. 2017), and the oral microbiome (Takayasu et al. 2017). It is essential to report on the use of saliva stimulation and enforce patient abstinence from food and drink for at least one hour prior to collection to ensure sample standardization. To prevent degradation and bacterial growth, collection on ice (4°C) is recommended (Bhattarai et al. 2018). Processing often includes sample dilution to reduce viscosity and improve isolation efficiency (Deregibus et al. 2016; Itzel et al. 2025; Kumeda et al. 2017; Reseco et al. 2024). Post‐isolation, salivary EVs demonstrate remarkable stability, remaining intact at 4°C for up to 20 months (Kumeda et al. 2017). Moreover, salivary EVs can be isolated from whole saliva that has been stored at 4 degrees for up to 28 days and still show intact membrane structure (Kumeda et al. 2017).

Beyond collection‐level controls, donor intrinsic biological variables also represent a source of pre‐analytical variability that requires attention. Oral health status can emerge as a meaningful confounder, given that conditions such as periodontitis and gingivitis alter both the cellular composition of the oral cavity and the local inflammatory milieu. This may have downstream consequences for the concentration and molecular cargo of salivary EVs. Gingival bleeding introduces erythrocyte and immune cell‐derived EVs into saliva, representing a contamination that may be difficult to distinguish from disease‐relevant signals without rigorous screening and reporting of participants’ periodontal status (Xu et al. 2025). Similarly, medications that affect salivary gland function (anticholinergics, antihypertensives, and antidepressants) can reduce salivary flow and alter protein secretion, introducing systematic confounding in studies that do not report or control for medication use (Ng et al. 2024).

The effects of freeze‐thaw cycles on salivary EV integrity constitute another critical, yet frequently overlooked, pre‐analytical variable. While a single freeze‐thaw cycle may exert insignificant effects on EV concentration and diameter (Kumeda et al. 2017; Yuana et al. 2015), repeated cycles significantly alter EV surface composition and membrane integrity. Consequently, if freezing is necessary, freezing and storage protocols must remain identical across comparative studies to ensure biorepository outputs remain reliable (Yuana et al. 2015). This consideration carries particular weight in multicentre studies and biobank‐based investigations, where identical freeze‐thaw histories across samples cannot always be guaranteed. Furthermore, achieving true methodological transparency requires investigators to report the comprehensive centrifugation history, from initial debris removal to final pelleting, rather than merely the final isolation step.

This lack of standardization across pre‐analytical and analytical workflows currently hinders the direct comparison of findings and results in highly variable reports of salivary EVs, where purity is often not reported as well. Bridging this gap requires a comprehensive reporting framework that includes oral health screening, medication documentation, demographic characteristics, freeze‐thaw history, and centrifugation parameters. Adopting such a framework is essential to achieve the reproducibility that clinical translation demands. Beyond reporting, the choice of isolation method must be strategically aligned with the study's objective, carefully balancing the competing demands of yield and purity. In this regard, hybrid methodologies such as combining high‐yield polymer‐based precipitation for enrichment with high‐purity SEC for final purification, offer a promising compromise. Ultimately, broad standardization efforts are essential to improve reproducibility and enable robust cross‐study comparisons in the salivary EV field.

## 4 Salivary Extracellular Vesicles in Disease Diagnostics

### 4.1 Neuropsychiatric and Neurodegenerative Disorders

**FIGURE 3:**
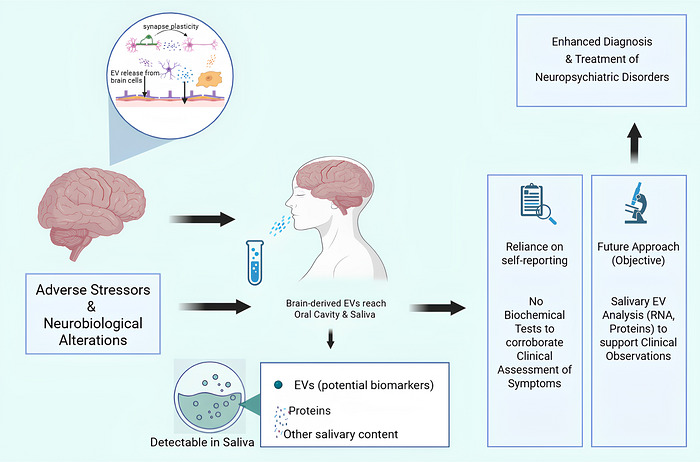
Salivary EVs as candidate diagnostic biomarkers for neuropsychiatric disorders. The schematic illustrates an emerging paradigm in which brain‐derived EVs, carrying neurobiological information, are hypothesized to reach the saliva and provide an accessible source of molecular biomarkers. Adverse stressors, and their associated neurobiological alterations, affect brain cell activity (including synaptic plasticity) and are accompanied by the release of EVs from brain cells (inset). These brain‐derived EVs are proposed to traffic from the central nervous system to the oral cavity, where they enter the saliva alongside proteins and other salivary constituents, becoming detectable in a readily accessible biofluid. The right‐hand panels contrast current clinical practice, which relies largely on subjective self‐reporting with no biochemical tests to corroborate symptoms‐based assessment, against a future objective approach in which salivary EV analysis (including RNA and protein cargo) supports clinical observation. Together, these elements point toward a more objective, biologically grounded diagnosis and treatment of neuropsychiatric disorders. Created in BioRender.

Novel avenues are increasingly being explored for the diagnosis of neuropsychiatric disorders. At present, the diagnosis and treatment of mental disorders rely predominantly on subjective clinical assessments of symptoms, with no validated biochemical tests to corroborate findings. Emerging evidence demonstrates that brain‐derived EVs can reach the oral cavity, and are detectable in saliva (Figure 3, Cheng et al. 2019; Rani et al. 2021). This observation supports the working theory that exposure to adverse stressors converges with measurable neurobiological alterations, which can be captured through objective diagnostic tools such as salivary EVs, thereby offering a means to substantiate clinical observations (Figure 3).

In this context, salivary EV contents have emerged as a promising, yet still exploratory, source of biomarkers for various neuropsychiatric disorders (Table 2). The research remains in a nascent phase; for example, Monteleone et al. (2020) identified salivary EV protein M6a (*n* = 88), a neuronal glycoprotein involved in differentiation, spine formation, and synaptogenesis, as a potential biomarker for depression and anxiety. Cohn et al. (2022) proposed salivary EV phosphoglycerate kinase 1 (PGK1) and miR‐3185 as markers for broad symptoms of mood variation and fatigue, with potential applications in risk assessment for reduced cognitive performance in occupations requiring sustained attention. More recently, Holliday et al. (2025) analysed salivary exosomal miRNAs to find objective indicators for high‐stress conditions to complement self‐reports obtained through the Adverse Childhood Experiences (ACE) survey and the Holmes and Rahe Social Readjustment Rating Scale (SRRS). They identified differentially expressed miRNAs in individuals with high chronic stress and/or high ACE scores, suggesting that these miRNAs may serve as objective indicators of psychological adversity (Holliday et al. 2025). Collectively, while these studies highlight the potential of salivary EV as objective tools for assessing mental status, they also underscore the field's current lack of convergence due to the paucity of studies investigating salivary EVs as biomarkers for neuropsychiatric conditions. Rather than reinforcing a single marker, these findings identify novel and distinct salivary EV molecular candidates and contribute to the growing body of evidence that supports salivary EVs as potential biomarkers for various conditions. However, no study has yet produced a marker with the specificity and validation required to supplant the traditional self‐reporting scales.

**TABLE 2:** Summary of published studies showing potential application of salivary EVs in neuropsychiatric conditions.

Condition	Biomarkers	Potential Application	Study Cohort	References
Depression & Anxiety	‐ OLIG2, PMP2, CNP, CAMK2A, SLC25A22, MLLT11, HTR2A, MAPT, ATP2B2, M6a	‐ Diagnosis of psychotic and depressive disorders, and guide therapeutic intervention	‐ 18 Healthy controls ‐ 70 Patients	(Monteleone et al. 2020)
Mood changes and Fatigue	‐ PGK1, miR‐3185	‐ Risk assessment for cognitive performance in attention‐demanding settings	‐ 36 Patients	(Cohn et al. 2022)
Chronic Stress and Childhood Trauma	‐ miR‐19b, miR‐187, miR‐34a, miR‐135‐3p	‐ Diagnostic and prognostic biomarkers for chronic stress and childhood trauma that could corroborate self‐reports of current evaluation scales.	‐ 12 Patients	(Holliday et al. 2025)
Alzheimer's Disease	‐ Olig‐amyloid β, p‐tau	‐ Early detection of AD	‐ 12 Healthy controls ‐ 17 AD patients	(Rani et al. 2021)
	‐ miR‐485‐3p	‐ Early detection of AD	‐ 13 Healthy controls ‐ 27 AD patients	(Ryu et al. 2023)
Traumatic Brain Injury	‐ ALOX5, ANXA3, CASP1, ITGB2, ADRB1, ADRB2, BDKRB1, CYSLTR1, HRH1, HRH2, LTB4R2, LTB4R, MC2R, NFKB1, PTAFR	‐ Diagnosis of TBI	‐ 7 Healthy controls ‐ 12 TBI patients	(Cheng et al. 2019a)
	‐ CDC2, CSNK1A1, CTSD	‐ Early detection of mild cases of TBI	‐ 23 Healthy controls ‐ 31 TBI patients	(Cheng et al. 2019b)
	‐ ALOX5, ITGB2, MAPK8, ANXA1, PDE4B, ADRB2, HRH1	‐ Early detection of TBI and provide quantitative driven return to play decisions in MMA	‐ 7 Healthy controls ‐ 20 MMA fighters	(Matuk et al. 2021)
Parkinson's Disease	‐ α‐synuclein	‐ Diagnostic biomarker for PD	‐ 60 Healthy controls ‐ 74 PD patients	(Cao et al. 2019)

Beyond psychiatric research, salivary EVs have also been identified as promising candidates for the diagnosis of neurodegenerative diseases such as Alzheimer's disease (AD). Rani et al. (2021) reported elevated salivary EV concentrations and increased expression of oligomeric amyloid‐β (Aβ) and phosphorylated tau (p‐tau) proteins in cognitively impaired individuals and AD patients. These findings were later corroborated by Ryu et al. (2023), who demonstrated that AD patients exhibit elevated concentrations of salivary EV miR‐485‐3p, which correlate positively with Aβ deposition in the brain (*r* = 0.6083, *p* < 0.0001) and have strong predictive value for Aβ‐PET positivity (AUC = 0.9217; sensitivity 86%, specificity 89%). Nonetheless, because AD is routinely diagnosed through categorical clinical judgement (based on composite criteria rather than single thresholds) it remains important to investigate how this salivary EV miRNA marker performs when applied using point‐biserial correlation with clinical diagnosis, as opposed to the biserial correlation applied by the investigators. Despite of challenges, biomarkers associated with salivary EVs hold a promising potential as less invasive and affordable diagnostic alternatives to the highly costing positron emission tomography (PET) imaging for Aβ deposition (Lee et al. 2021).

Similarly, traumatic brain injury (TBI), a known risk factor for AD due to post‐traumatic inflammation, has also been linked to distinct salivary EV signatures. Cheng et al. (2019) found that EVs from brain trauma patients exhibited upregulation of genes associated with neural plaque formation, amyloid precursor proteins, and apoptosis. Genes implicated in neuroinflammation and AD development, including CTSD, CASP1, CSNK1A1, and TNFRSF1A, were consistently upregulated (Cheng et al. 2019a; Cheng et al. 2019b). Furthermore, the same research group reported salivary EV gene profiles that closely resembled those observed in mixed martial arts (MMA) fighters, a high‐impact sport with significant TBI risk (Cheng et al. 2019b; Matuk et al. 2021). Genes such as ALOX5, ITGB2, ADRB2, and HRH1 were upregulated both in patients with acute concussive injury (within 24 hours) and in MMA fighters one‐hour post‐fight, as well as in individuals with chronic TBI exposure (concussion clinic patients and pre‐fight MMA samples). These genes function as mediators of cellular stress response, inflammation regulation, and signal transduction. The findings underscore the role of EVs as sensitive biomarkers not only in severe cases but also in mild TBI, which are often overlooked despite their potential contribution to AD pathogenesis.

A similar diagnostic challenge exists in Parkinson's disease (PD), which is difficult to differentiate clinically from atypical parkinsonian syndromes due to overlapping symptoms despite distinct underlying pathologies. Mitochondrial impairment is central to PD pathophysiology, particularly in the formation of Lewy bodies, where α‐synuclein plays a critical role. In pursuit of non‐invasive diagnostic tools, Cao et al. (2019) demonstrated significantly higher levels of oligomeric α‐synuclein (α‐synolig) and α‐synolig/α‐synTotal in salivary EVs of PD patients compared to healthy controls (10.39 pg/ng vs. 1.37 pg/ng and 1.70 pg/ng vs. 0.67 pg/ng, respectively). Although both markers successfully distinguished PD patients from controls, no correlation was established between elevated marker levels and clinical parameters such as the Hoehn and Yahr scale.

These studies offer compelling evidence that establishes salivary EVs as prominent and encouraging source of non‐invasive and objective biomarkers for neuropsychiatric and neurodegenerative disorders. The findings collectively demonstrate that brain‐derived EVs are detectable in the saliva, and they carry specific molecular cargo reflecting neurobiological alterations underlying conditions of the nervous system. This consensus reinforces the proof‐of‐concept, demonstrating a significant paradigm shift in a field that has historically relied on subjective and invasive methods of diagnosis. However, this is not without significant challenges in translating discovery into clinical practice. The current studies reveal a critical gap between biomarker identification and practical application. Therefore, future research must prioritize a pivot from discovery to rigorous clinical validation and standardization of isolation protocols to fully realize the potential of accessible and affordable diagnostic tools through salivary EVs.

### 4.2 Cancer

Cancer remains a major global health burden and is among the leading causes of death from non‐communicable diseases. Lung cancer, in particular, is a dominant contributor to both morbidity and mortality, accounting for 12.4% of cases and 18.7% of cancer‐related deaths worldwide (Bray et al. 2024). Salivary EVs have been highlighted as potential carriers of tumor‐specific biomarkers including lung, head and neck and other cancers (Table 3). Salivary EVs could play a crucial role in early detection and monitoring of head and neck cancers, as well as cancers from distal organs (Cui et al. 2024).

**TABLE 3:** Salivary EVs‐associated biomarkers for cancer diagnosis, prognosis, and treatment monitoring.

Cancer type	EV‐associated biomarkers	Biomarker potential	Study cohort	Reference
Lung cancer	‐ IGLC7, VIM, PLTP CRA_c, Annexin, AZGP1, LPO, PSMA6, GCA, CRISP3, CAPNS1, H3F3B	‐ Diagnostic.	‐ 3 Healthy controls ‐ 3 Lung cancer patients	(Sun et al. 2017)
	‐ BPIFA1, CRNN, MUC5B, IQGAP	‐ Diagnostic.	‐ 6 Healthy controls ‐ 6 Lung Cancer patients	(Sun et al. 2018)
	‐ SPARC‐like protein 1, EPS8 protein 2, ANO1 protein	‐ Diagnostic.	‐ 18 Healthy controls ‐ 6 Lung Cancer patients	(Wahid et al. 2022)
	‐ miR‐92b‐5p, miR‐135b‐5p, miR‐532‐3p	‐ Diagnostic.	‐ 18 non‐cancer subjects ‐ 18 Lung cancer patients	(Liu et al. 2023)
Oral squamous	‐ miR‐412‐3p, miR‐512‐3p, miR‐27a‐3p, miR‐373‐3p, miR‐494‐3p, miR‐302b‐3p, miR‐517b‐3p	‐ Diagnostic. ‐ Prognostic.	‐ 11 Healthy controls ‐ 21 OSCC patients	(Gai et al. 2018)
	‐ miR‐140‐5p, miR‐143‐5p, miR‐145‐5p	‐ Diagnostic. ‐ Prognostic.	‐ 3 Healthy controls ‐ 28 OSCC patients	(Patel et al. 2023)
	‐ miR‐486‐5p, miR‐10b‐5p	‐ Diagnostic	‐ 25 Healthy controls ‐ 25 OSCC patients	(Faur et al. 2022)
	‐ miR‐1307‐5p	‐ Prognostic	‐ 8 Healthy controls ‐ 20 OSCC patients	(Patel et al. 2022)
	‐ G‐*N*chiRNA	‐ Diagnostic ‐ Prognostic	‐ 125 Healthy controls ‐ 419 OSCC	(Lin et al. 2019)
	‐ tRNA‐GlyGCC‐5, sRESE	‐ Diagnostic ‐ Prognostic	‐ 36 Healthy controls ‐ 36 OSCC patients	(Li et al. 2022)
	‐ AMER3, LOXL2, AL9A1, PSB7	‐ Diagnostic	‐ 20 Healthy controls ‐ 10 OSCC patients ‐ 20 OPMD	(Bozyk et al. 2023)
	‐ cirPRMT5	‐ Diagnostic	‐ 29 Healthy controls ‐ 72 OSCC	(Jiang et al. 2025)
	‐ mRNAs of TNF‐α, MMP9 and OAZ1	‐ Diagnostic. ‐ Prognostic.	‐ 40 Healthy Control. ‐ 40 OSCC patients.	(Kumar et al. 2025)
HPV‐driven oropharyngeal cancer	‐ ALDOA, GAPDH, LDHA, LDHB, PGK1, PKM	‐ Diagnostic	‐ 20 Healthy controls ‐ 10 OSCC patients	(Tang et al. 2021)
Prostate cancer	‐ hsa‐mir‐200b and hsa‐mir‐331‐3p	‐ Diagnostic	‐ 31 non‐cancer patients ‐ 43 Prostate cancer patients	(Luedemann et al. 2022)
Pancreatobiliary tract cancer	‐ miR‐1246 and miR‐4644	‐ Diagnostic	‐ 13 Healthy control ‐ 12 Cancer patients	(Machida et al. 2016)

#### 4.2.1 Lung Cancer

The immune system functions as a double‐edged sword in the context of cancer. While it can suppress tumor development, it can also promote tumor progression by dampening anti‐tumor immunity and facilitating chronic inflammation; EVs are known to play a role in this modulation (Kuang et al. 2025; Shurin 2012; Zamarron and Chen 2011). Cancer‐derived EVs carrying oncogenic cargo have been implicated in promoting the growth, progression, and metastasis of tumours. Consequently, recent efforts have focused on easily accessible biofluids, such as saliva, to isolate EVs and identify associated biomarkers for early diagnosis and prognosis of lung cancer.

Sun et al. (2017) examined the proteome of exosomes from saliva and serum of lung cancer patients and found a high degree of overlap, with 80% of proteins shared between the two biofluids. This finding demonstrated saliva's potential to serve as a “mirror” of systemic conditions. In this study, salivary EV proteomic profiling identified 29 proteins associated with lung cancer that are directly linked to the Akt/MAPK and RAS‐MAPK pathways, which are central to cancer proliferation and metastasis. Building on this work, Sun et al. (2018) further analyzed salivary EV proteomes of lung cancer patients and identified six highly dysregulated proteins. Four of these, CRNN, MUC5B, BPIFA1, and IQGAP1, were confirmed by immunoblotting and are mediators of proliferation and apoptosis in non‐small cell lung cancer. Crucially, these proteins showed no significant differences in whole saliva between cancer patients and healthy controls, underscoring that their diagnostic potential is specific to the EV fraction rather than the whole saliva.

**FIGURE 4:**
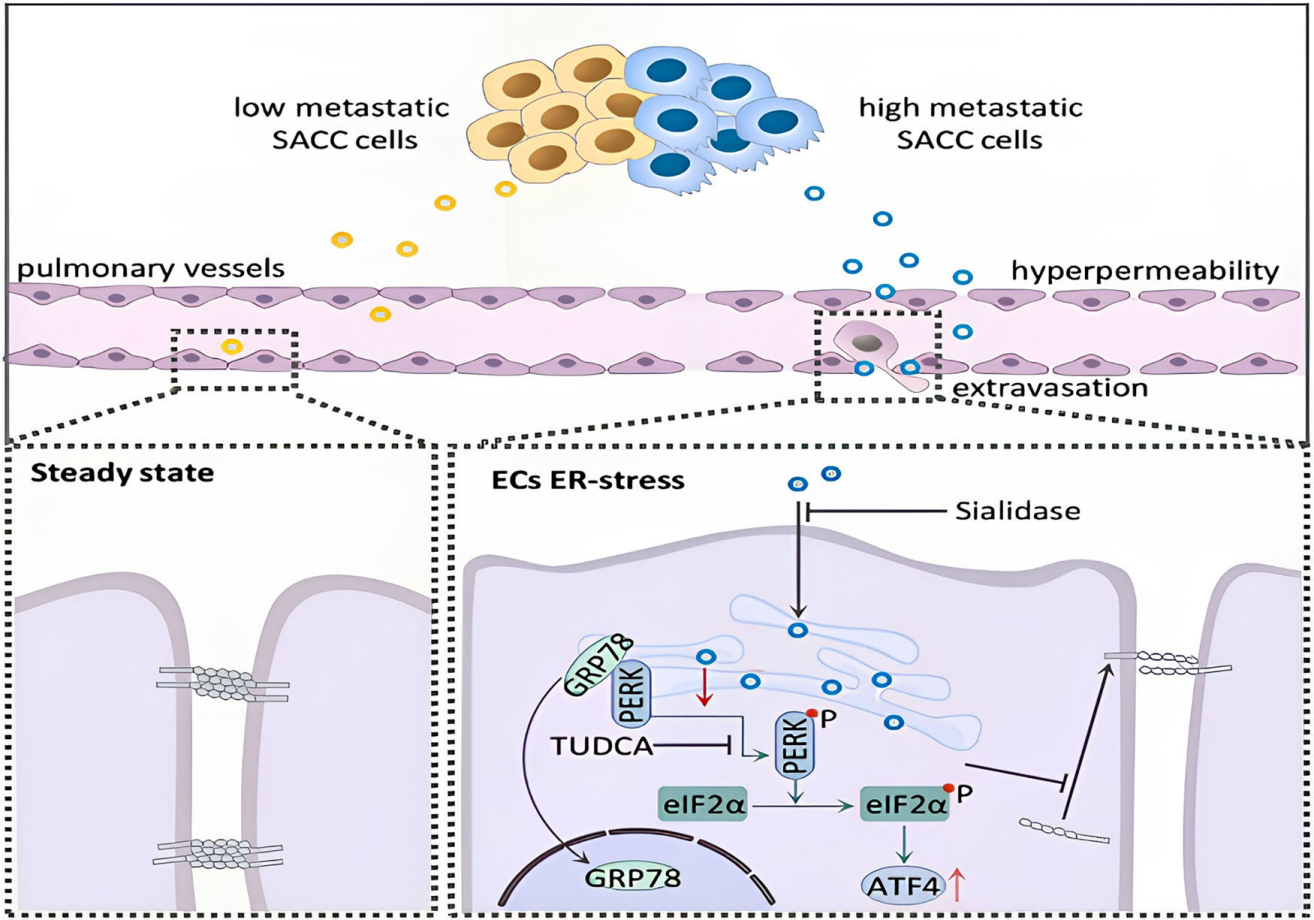
Salivary EV α2,6‐sialylation induces vascular hyperpermeability by triggering endoplasmic reticulum (ER) stress in endothelial cells. Salivary adenoid cystic carcinoma (SACC) cells of low and high metastatic potential release EVs into the pulmonary vasculature (top). EVs derived from highly metastatic SACC cells carry α2,6‐sialylated surface proteins and increase endothelial permeability, promoting tumour cell extravasation into lung tissue. The lower panels contrast the endothelial barrier under steady‐state conditions, in which intercellular junctions remain intact, with its response to α2,6‐sialylated EVs (ECs ER‐stress). Internalised EVs localise to the ER and activate the PERK‐eIF2α‐ATF4 arm of the unfolded protein response: protein kinase R‐like ER kinase (PERK) is phosphorylated (P), which in turn phosphorylates eukaryotic initiation factor 2α (eIF2α) and upregulates activating transcription factor 4 (ATF4), alongside induction of the ER chaperone GRP78. This signalling disrupts endothelial intercellular (VE‐cadherin) junctions, increasing vascular permeability. Two interventions are shown: sialidase, which removes the EV α2,6‐sialylation, and tauroursodeoxycholic acid (TUDCA), an ER‐stress inhibitor that blocks PERK activation; both attenuate the response. Reprinted from Dong et al. (2025), with permission from Elsevier [License number: 6279580393786].

In addition to protein markers, salivary EV cargo also reflects changes in post‐translational modifications. Dysregulated phosphoproteins have been detected in salivary EVs, offering another layer of biomarker potential. Wahid et al. (2022) employed Ti4+ immobilized affinity chromatography to profile salivary EV phosphoproteins and identified 32 significantly dysregulated proteins in lung cancer patients, including SPARC‐like protein 1, EPS8‐related protein 2, and anoctamin 1 (ANO1), all of which are implicated in non‐small cell lung cancer. Similarly, genomic studies highlight salivary EV microRNAs (miRNAs) as promising candidates for liquid biopsy. Liu et al. (2023) identified three significantly upregulated miRNAs (*n* = 36; miR‐92b‐5p, miR‐135b‐5p, and miR‐532‐3p) in saliva‐derived EVs from lung cancer patients. This signature of miRNAs is associated with cancer, with miR‐92b‐5p implicated in gastric and biliary tract cancers (Høgdall et al. 2022; Xu et al. 2021). These miRNAs achieved high diagnostic accuracy (AUC 0.912; *p* < 0.001) in distinguishing cancer from healthy controls, further supporting the utility of biomarker panels over single markers. Mechanistic studies also link salivary EVs to cancer *metastasis*. Dong et al. (2025) demonstrated that salivary EV proteins promote the formation of a pre‐metastatic niche via α2,6‐sialylation–induced endoplasmic reticulum stress in endothelial cells (Figure 4). This process increases vascular permeability, enabling salivary gland adenoid cystic carcinoma cells to extravasate into the lungs. Collectively, these studies highlight the translational promise of salivary EVs as liquid biopsy tools, capable of reflecting both local and systemic disease states.

The research in lung cancer shows a significant translational promise through the identification of EV biomarkers across the proteomic, phosphoproteomic, and genomic modalities. These studies show that saliva can mirror systemic conditions and that specific enrichment of salivary EVs is necessary to identify the dysregulated signals that would otherwise not be detected in whole saliva. However, the findings of several studies are divergent and predominantly discovery as they identify novel and often non‐overlapping sets of biomarkers. Therefore, future work must focus on large scale validation to converge on a consensus panel and illuminate on specific pathways by which distant tumours transport biomarkers to the oral cavity.

#### 4.2.2 Head and Neck Carcinomas

Head and neck squamous cell carcinoma (HNSCC), particularly oral squamous cell carcinoma (OSCC), is among the most common malignancy in this region, accounting for 4.5% of cancer diagnoses and 4.6% of cancer‐related deaths globally (Fatima et al. 2024). These tumours arise from the mucosal epithelium of the oral cavity, larynx, or pharynx. Despite advances, early diagnosis remains a significant challenge; however, it is crucial for improving patient survival and reducing surgical burden. Histopathological staging remains the gold standard for OSCC management, but there is a growing demand for more efficient, non‐invasive predictive factors for diagnosis, prognosis, and monitoring therapeutic response.

Salivary EV–derived miRNAs are particularly promising biomarkers for early OSCC detection, as diagnosis currently relies on tissue biopsy and physical examination. Multiple studies have uncovered salivary EV miRNA signatures associated with OSCC. Gai et al. (2018) reported upregulation of miR‐412‐3p, miR‐512‐3p, miR‐27a‐3p, miR‐373‐3p, and miR‐494‐3p, with miR‐302b‐3p and miR‐517b‐3p uniquely expressed in OSCC‐derived salivary EVs. mir‐512‐3p and mir‐412‐3p displayed high AUC scores of 0.847 and 0.871 respectively, both with *p* values < 0.02. Notably, the functions of several of these miRNAs in OSCC remain uncharacterized, underscoring the need for further validation. In contrast, Patel et al. (2023) identified a three‐miRNA signature—miR‐140‐5p, miR‐143‐5p, and miR‐145‐5p, that was significantly downregulated in OSCC salivary exosomes. This signature achieved high diagnostic accuracy (AUC 0.99, *p* < 0.0001, 98% sensitivity, and 99% specificity), outperforming tissue‐based analysis. Target gene analysis revealed HIF1A and CDH1 as central hubs, implicating dysregulation of epithelial–mesenchymal transition (EMT) pathways in OSCC progression.

Beyond diagnostic accuracy, salivary EV miRNAs can also serve as site‐specific and stage‐specific biomarkers. For example, overexpression of miR‐486‐5p was shown to distinguish oropharyngeal from oral cavity cancers, achieving an AUC of 0.89 versus 0.67, respectively (Faur et al. 2022). The same miRNA was elevated in stage II oral and oropharyngeal cancers compared to later stages, suggesting stage‐dependent expression.

Salivary EV biomarkers also hold promise for real‐time monitoring and prognosis, overcoming the limitations of tissue biopsy. Patel et al. (2022) proposed salivary EV miR‐1307‐5p as a prognostic biomarker, as its elevated expression correlated with poor survival, aggressive disease, and therapeutic refractoriness. Similarly, Lin et al. (2019) reported that salivary EV G‐NchiRNA, a hybrid transcript derived from GOLM1 and NAA35 genes, displayed high sensitivity (89%) and specificity (91%) for detecting oesophageal squamous cell carcinoma (ESCC) and accurately predicted progression‐free survival and treatment response. A multicentre study by Li et al. (2022) further identified a salivary exosomal tRNA bi‐signature (tRNA‐GlyGCC‐5 and sRESE), with diagnostic sensitivity of 90.5% and specificity of 94.2% in distinguishing ESCC patients from healthy individuals. These RNAs were also detected in ESCC tissue and cell lines, supporting their tumor origin and biological relevance.

**FIGURE 5:**
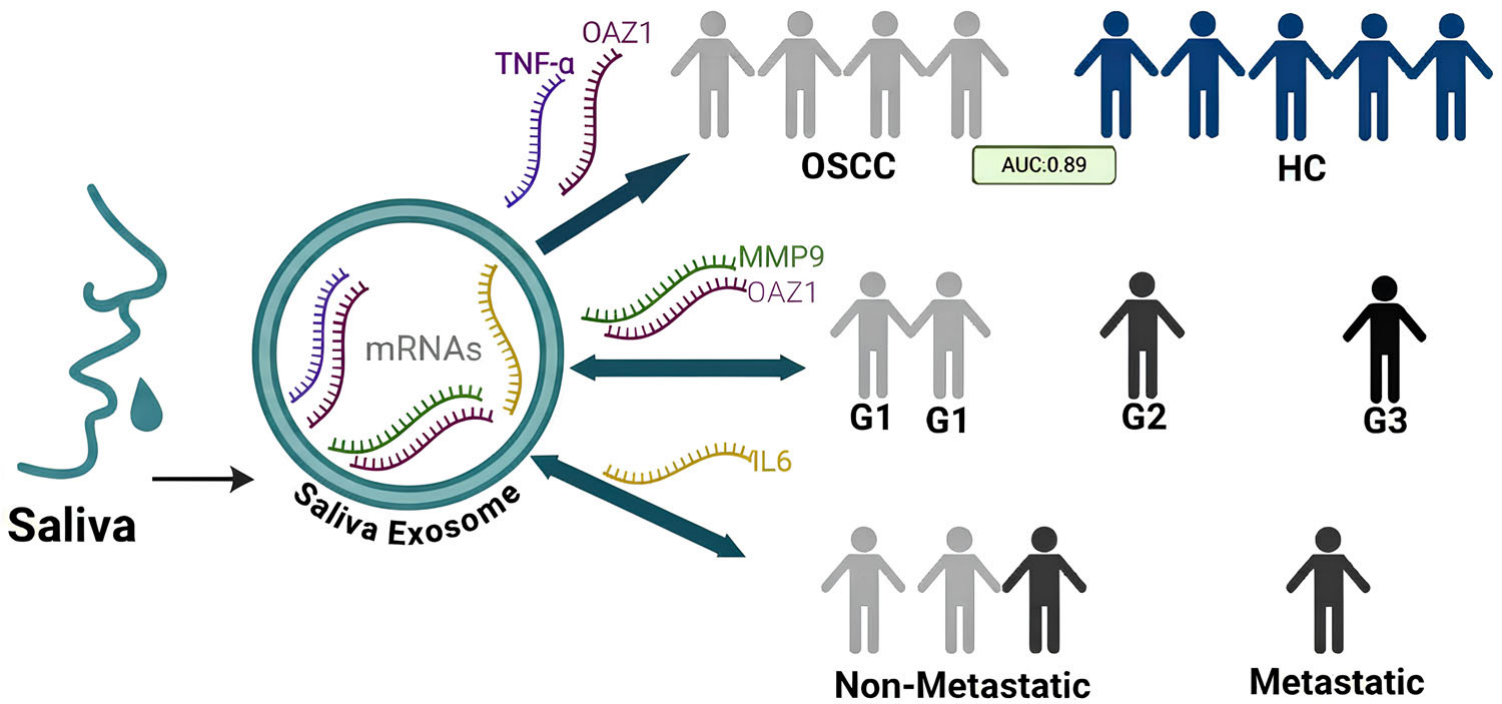
Diagnostic and prognostic potential of salivary exosome‐derived mRNAs in oral squamous cell carcinoma (OSCC). Saliva‐derived EVs carry mRNA cargo that discriminates disease states across three clinical axes. For diagnosis, a two‐marker panel of TNFα and OAZI distinguishes OSCC patients from healthy controls (HC), with an area under the ROC curve (AUC) of 0.89. For prognosis, OAZI, together with MMP9, associates with histological tumour grade, expression rising from grade 1 (G1) through grade 3 (G3). Elevated IL‐6 correlates with lymph node metastasis, separating metastatic from non‐metastatic disease. Collectively, these salivary EV mRNA signatures support a non‐invasive approach to OSCC detection, grading, and metastatic‐risk assessment. Reproduced from Kumar et al. (2025) under the terms of the Creative Commons Attribution license.

Salivary EV mRNA profile has also been recently investigated by Kumar et al. (2025) for the detection of OSCC using a two‐gene panel of TNF‐α and OAZ1with AUC of 0.89, sensitivity of 80%, specificity of 90% and Youden Index of 0.70 (Figure 5; Kumar et al. 2025). Furthermore, the study demonstrated the prognostic potential of salivary EV transcripts, as MMP9 and IL6 correlated significantly with tumour grade and lymph node metastasis respectively, a discriminatory power that serum small EVs lacked (Figure 5, Kumar et al. 2025). This pilot study conducted on 80 participants (40 OSCC patients and 40 healthy controls) utilized a paired‐sample approach (serum and saliva) but with inherent bias of a candidate‐gene panel compared to a broader discovery approach. Critically, future research should include a control group of leukoplakia (benign oral lesions) to validate the diagnostic specificity of the proposed markers.

Proteomic profiling of salivary EVs provides a complementary approach to cancer diagnostics. Bozyk et al. (2023) reported several differentially expressed proteins with diagnostic potential in OSCC, including AMER3, LOXL2, AL9A1, and PSB7. AL9A1, an isoform of ALDH, is known to contribute to solid tumour development whereas AMER3 is a positive regulator of the Wnt signalling pathway and is linked to colorectal cancer and OSCC development (Brauburger et al. 2014; Xie et al. 2021). LOXL2 is implicated in promoting cancer cell proliferation, migration, invasion, and metastasis, and PSB7 is involved in the regulation of protein homeostasis and associated with poor prognosis of breast cancer (Cano et al. 2012; Tian et al. 2019; Yoon et al. 2020). The use of a three‐protein panel of PSB7, AMER3, and LOX12 had an AUC of 0.96, sensitivity of 100% and specificity of 75%. In a separate study, Tang et al. (2021) demonstrated that human papilloma virus (HPV)‐driven oropharyngeal cancers exhibited elevated levels of six glycolytic enzymes (ALDOA, GAPDH, LDHA, LDHB, PGK1, and PKM) in salivary EVs, reflecting HPV's role in enhancing the Warburg effect and shaping a tumour‐permissive microenvironment. LDHB had the highest AUC of 0.93 (*p* = 0.0002; 95% CI: 0.8425–1.000) whereas PGK1 had the lowest AUC of 0.73 (p = 0.043; 95% CI: 0.5499–0.9101) (Tang et al. 2021). More recently, Jiang et al. (2025) identified circPRMT5 as highly upregulated in salivary exosomes from HNSCC patients. Functionally, circPRMT5 promoted tumour progression via the IGF2BP3–SERPINE pathway, and its salivary EV levels decreased after tumour resection, supporting its potential as a diagnostic biomarker and therapeutic target.

#### 4.2.3 Prostate and Pancreatic Cancer

Salivary EVs are also promising candidates for the detection of cancers of distant organs since the saliva offers an accessible and non‐invasive diagnostic medium. A major limitation of the current prostate cancer diagnostics, PSA testing and transrectal biopsy, is their low predictive value, poor sensitivity, and invasiveness (Ilic et al. 2018). Addressing this gap, Luedemann et al. (2022) analysed 16 prostate cancer‐associated miRNAs in salivary EVs from 74 male participants using the delta‐CT method. Among these, hsa‐mir‐200b and hsa‐mir‐331‐3p showed significant differential expression between prostate cancer and non‐cancer groups, yielding a positive predictive value of 71%. Both miRNAs demonstrated a moderate but reliable discriminatory power (AUC = 0.663 for hsa‐mir‐200b and 0.648 for hsa‐mir‐331‐3p), despite the comparison being limited to prostate cancer patients and non‐cancer individuals with elevated PSA levels. Encouragingly, these results suggest a robustness in the population with reduced spectrum bias; however, validation using a healthy control group and larger sample size is required to establish diagnostic performance relative to PSA.

In another study, Machida et al. (2016) investigated salivary EV miRNAs as biomarkers of pancreatobiliary tract cancer. Using qRT‐PCR, they identified two miRNAs (miR‐1246 and miR‐4644) that were significantly upregulated in cancer patients (*n* = 12) compared with healthy individuals (*n* = 13). Diagnostic analysis showed that miR‐1246 achieved an AUC of 0.814 (95% CI: 0.616–1.00; sensitivity 0.667; specificity 1.00; *p* = 0.008), while miR‐4644 had an AUC of 0.763 (95% CI: 0.564–0.961; sensitivity 0.750; specificity 0.769; *p* = 0.026) (Machida et al. 2016). Combined, they yielded an improved AUC of 0.833 (*p* = 0.005), underscoring the enhanced diagnostic value of multiplexed biomarkers over single markers. To facilitate clinical translation, larger multi‐centre studies incorporating patients with pancreatitis and early‐stage pancreatobiliary cancer are needed to validate these findings and assess inter‐individual variability.

### 4.3 Salivary Extracellular Vesicles in Sjogren Syndrome

Primary Sjögren syndrome (pSS) is a chronic systemic rheumatic autoimmune disorder characterized by the presence of autoantibodies against SSA/Ro and SSB/La ribonucleoproteins particles, as well as mononuclear cell infiltration of exocrine tissues (Jonsson et al. 2007). Keratoconjunctivitis sicca and xerostomia are hallmark clinical features of this disease. The condition typically begins around the age of 45–55 years and affects approximately 0.5%–1% of the population, with an estimated ∼3 million individuals living with the disease (Carsons and Blum 2025). A major challenge in diagnosing pSS is that significant glandular dysfunction is already present at the time of clinical diagnosis, implying that autoimmune processes are underway long before symptom onset. This is evident in the diminished glandular function that manifests as dry mouth and dry eyes. Moreover, current diagnostic approaches rely on invasive procedures, such as salivary gland tissue biopsy and the detection of anti‐SSA/Ro antibodies, to confirm diagnosis (Wang et al. 2021). Therefore, early, non‐invasive biomarker strategies are needed to preserve residual glandular function. Potential biomarkers for pSS and other systemic conditions are summarized in Table 4.

**TABLE 4:** Salivary EVs‐associated biomarkers for diagnosis, prognosis, and treatment monitoring of systemic diseases other than cancer.

Disease	Biomarkers	Biomarker Potential	Study Cohort	Reference
Primary Sjogren Syndrome (pSS)	‐ APMAP, GNA13, WDR1	‐ Diagnostic	‐ 32 Healthy controls ‐ 27 pSS patients	(Aqrawi et al. 2017)
	‐ LCN2, CD44	‐ Diagnostic	‐ 10 Healthy controls ‐ 10 pSS patients ‐ 15 non‐pSS patients	(Aqrawi et al. 2019)
	‐ tRNA‐Ile‐AAT‐2‐1	‐ Diagnostic	‐ 11 Healthy controls ‐ 11 pSS patients	(Cross et al. 2023)
Inflammatory Bowel Disease (IBD)	‐ PSMA7	‐ Diagnostic	‐ 10 Healthy controls ‐ 48 IBD patients	(Zheng et al. 2017)
	‐ hsa‐mir‐1246, hsa‐mir‐142‐3p, hsa‐mir‐16‐5p, hsa‐mir‐301a‐3p, hsa‐mir‐4516	‐ Diagnostic	‐ 24 Healthy controls ‐ 126 IBD patients	(Yang et al. 2025)

**FIGURE 6:**
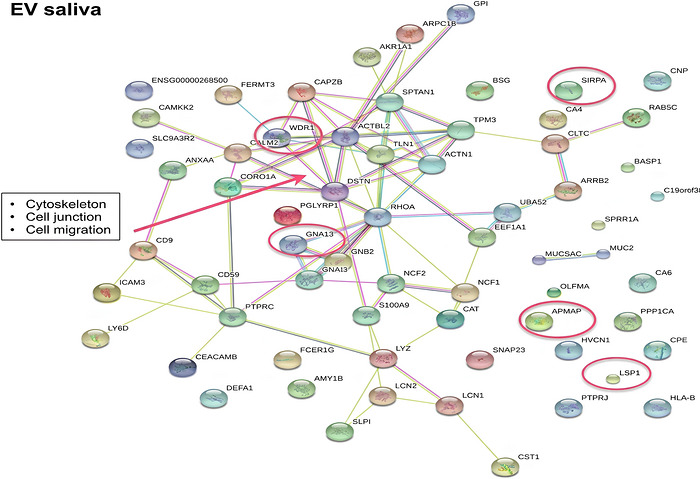
Protein–protein interaction (PPI) network of upregulated salivary EV proteins in primary Sjögren's syndrome (pSS). The network was generated in STRING, in which nodes represent proteins and connecting edges denote known and predicted functional associations. Five upregulated salivary EV proteins of particular interest are highlighted (red circles): SIRPA, WDR1, GNA13, APMAP, and LSP1. SIRPA (signal regulatory protein α) and LSP1 (lymphocyte‐specific protein 1) contribute to activation of the innate immune system; APMAP (adipocyte plasma membrane‐associated protein) is an enzyme involved in adipocyte differentiation, GNA13 (guanine nucleotide‐binding protein subunit α‐13) participates in signal transduction; and WDR1 (WD repeat‐containing protein 1) regulates actin‐filament turnover relevant to cell migration and chemotaxis. These proteins are associated with cytoskeletal organisation, cell junctions, and cell migration. Adapted from Aqrawi et al. (2017) under the terms of the Creative Commons Attribution license.

Several studies have investigated salivary EVs as potential biomarker reservoirs for pSS. Proteomic profiling of salivary EVs from pSS patients revealed upregulation of APMAP, GNA13, and WDR1 as illustrated in Figure 6 (Aqrawi et al. 2017). APMAP is an enzyme implicated in adipocyte development and immune regulation, while GNA13 and WDR1 are associated with inflammatory processes and tissue damage. These proteins were proposed as potential screening biomarkers, offering additional diagnostic accuracy. Furthermore, Aqrawi et al. (2019) reported that CD44 and lipocalin‐2 (LCN2)—an iron‐binding protein that mediates innate immune responses and neutrophil activation, were consistently upregulated in whole saliva and salivary EVs of pSS patients compared to healthy controls and non‐pSS sicca patients, across independent cohorts. These findings position LCN2 as a strong candidate biomarker for pSS.

Beyond proteins, non‐coding RNAs within salivary EVs have also been explored. Cross et al. (2023) identified downregulation of tRNA‐Ile‐AAT‐2‐1 in salivary EVs of pSS patients, suggesting its utility as a potential biomarker. Additionally, salivary EV RNA cargo has been investigated for therapeutic applications. Xie et al. (2025) demonstrated that EVs derived from labial gland mesenchymal stem cells (MSCs), transfected with let‐7f‐5p, were able to suppress proinflammatory Th17 cell activity and restore the Th17/Treg balance. Mechanistically, let‐7f‐5p‐enriched EVs inhibited the RORC/IL‐7A signalling axis, thereby restoring salivary flow and reducing lymphocytic infiltration.

Beyond their diagnostic potential, the therapeutic application of EVs in pSS has also been demonstrated clinically. In a randomized trial, human Wharton's jelly MSC‐derived EVs formulated as topical eye drops showed significant benefits in pSS patients with dry eye (Habibi et al. 2025). Treatment reduced expression of proinflammatory mediators (IL‐6, IL‐22, and MMP‐9) while upregulating multifunctional proteins such as EGF, LTF, and THBS1, which are key mediators of tissue repair. Collectively, these studies highlight the diagnostic and therapeutic potential of salivary EVs in pSS. While current therapy remains largely symptomatic, EV‐based approaches offer promising avenues for earlier detection and targeted treatment of this debilitating autoimmune disorder.

### 4.4 Salivary EVs and other systemic conditions

Extracellular vesicles are key mediators in numerous biological processes including haemostasis. Their procoagulant activity is primarily attributed to tissue factor (TF) and activated factor VIIa (FVIIa) on their surfaces, forming the extrinsic tenase complex. The detection of TF‐bearing EVs in various biofluids such as amniotic fluid, milk, saliva, and urine has diverse physiological and pathological implications. These include their proposed involvement in the pathogenesis of amniotic fluid embolism‐induced disseminated intravascular coagulation (AFE‐Induced DIC) (Hell et al. 2017), the protection of infant gut integrity by limiting gastrointestinal bleeding (Hu et al. 2020), and the mitigation of oropharyngeal or wound bleeding triggered by wound‐licking reflex (Berckmans et al. 2011).

The hypothesis that body fluids exposed to the ‘*milieu extérieur*’ contain TF‐bearing EVs that contribute to wound healing provides a plausible explanation for the wound‐licking reflex. Supporting this, Berckmans et al. (2011) demonstrated the procoagulant capacity of these TF‐bearing salivary EVs, showing a significant reduction in clotting time from 300 ± 96 to 186 ± 24 s (*p* = 0.03) in wound blood. These findings suggest that the reflex represents an evolutionary conserved immune defence mechanism in humans and other animals. Expanding on this, Yu et al. (2018) showed that salivary EVs colocalize TF and CD24, promoting fibrin generation through binding and accumulation on activated platelet surfaces. Collectively, these observations indicate that salivary EVs are functionally adapted for oral cavity immunity, acting as a first line of defence against both external pathogens and the resident microbiota.

Building on this, Hu et al. (2022) examined the contributions of EVs containing extrinsic tenase complex in haemostatic protection. Using saliva and other biofluids, they demonstrated that EV‐rich fractions, rather than EV‐depleted counterparts, effectively promoted FXa generation in both normal plasma (TF deficient) and FVIIa deficient plasma (TF and FVIIa deficient). Similar findings were reported by Thaler et al. (2024), who confirmed the presence of extrinsic tenase complex in salivary EVs of persons with haemophilia A, suggesting a role for these EVs in reducing the frequency of oropharyngeal bleedings in this patient population. Moreover, they showed that individuals with severe FVII deficiency produce salivary EVs lacking functional extrinsic tenase complexes, offering a mechanistic explanation of the differing bleeding phenotypes between severe haemophilia A and severe FVII deficiency. These findings underscore the importance of salivary EVs as functional contributors to haemostasis in both physiological and pathological contexts. In bleeding disorders such as haemophilia A, insights into the coagulant properties of salivary EVs enhance the understanding of site‐specific bleeding tendencies and provide additional evidence for localized mechanisms of oropharyngeal coagulation.

A seminal study by Lau et al. (2013) further explored the role of salivary EVs in systemic disease biomarker development using orthotopic, syngeneic pancreatic cancer mouse model. They identified and validated seven (Apbb1ip, Aspn, Incenp, Daf2, Foxp1, BC031781, and Gng2) significantly upregulated in saliva of tumour‐bearing mice, five of which were enriched within saliva‐derived EVs. This discriminatory transcriptomic profile supported a mechanistic model wherein distal tumour cells communicate with oral cavity via exosomes to induce disease‐specific salivary markers. This hypothesis was partly supported by their earlier in vitro breast cancer study showing that tumour‐derived EVs could alter the composition of EVs secreted by salivary glands (C. S. Lau and Wong 2012). To test this mechanistic link, they inhibited pancreatic tumour exosome release by orthotopically injecting Panc02 cells expressing DN‐Rab11 (a dominant‐negative GTPase that supresses the biogenesis of exosomes). This disruption abrogated the established seven‐gene signature, leaving only Apbb1ip significantly upregulated (Lau et al. 2013). However, the study could not determine the precise mechanism of communication between distal tumours and the oral cavity. Potential explanations may include the transport of distal EVs through the lymphatic or circulatory pathways, filtration into the oral cavity via GCF, or translocation through the salivary gland vasculature. Human studies are needed to validate and elucidate these mechanisms.

Inflammatory bowel disease (IBD), encompassing ulcerative colitis and Crohn's disease, is a chronic immune‐mediated disorder of the gastrointestinal tract with unclear aetiology. Given the oral‐gut axis, salivary EVs have emerged as potential non‐invasive biomarkers for IBD, potentially sparing patients from invasive colonoscopies and biopsies. Zheng et al. (2017) analysed the salivary EV proteome using shotgun mass spectroscopy and identified significantly elevated levels of proteasome subunit alpha type 7 (PSMA7) in IBD patients (*n* = 48) compared to healthy controls (*n* = 11). PSMA7 is implicated in immune modulation and inflammation, though the mechanistic link between salivary EV PSMA7 and IBD pathogenesis remains to be established. The interplay between intestinal inflammation and oral manifestations in IBD is well documented (Lauritano et al. 2019; Ray 2020; Rogler et al. 2021). In a study employing the dextran sulphate sodium (DSS) mouse model, Yang et al. (2024) demonstrated that salivary EVs from patients with active IBD induced mucosal inflammation and epithelial injury in mice, characterized by M1 macrophage infiltration. In contrast, EVs from patients in remission and healthy controls did not elicit this response. Mechanistically, IBD‐associated salivary EVs activated the NF‐κB pathway by promoting the degradation of the inhibitory protein IκB‐α, thereby facilitating nuclear translocation of NF‐κB (p56) and transcription of downstream target genes (Yang et al. 2024). Moreover, these EVs contained 29 upregulated miRNAs, suggesting a regulatory role in inflammation (Yang et al. 2024). A follow‐up genetic study (Yang et al. 2025) identified a five‐miRNA diagnostic signature (hsa‐mir‐1246, hsa‐mir‐142‐3p, hsa‐mir‐16‐5p, hsa‐mir‐301a‐3p, and hsa‐mir‐4516) with potential clinical utility for IBD diagnosis and monitoring. The composite model validated in an independent cohort (*n* = 120) achieved an AUC of 1.00 for distinguishing IBD patients from healthy controls and 0.86 for differentiating active disease from remission (Yang et al. 2025). Remarkably, the miRNA profiles reported by (Yang et al. 2025) largely overlapped with those observed in their 2024 cohort, indicating strong inter‐study concordance. Nevertheless, further preclinical validation using standardized salivary EV isolation methods and larger, diverse cohorts is essential to enhance reproducibility and facilitate clinical translation.

## 5 Conclusion

Salivary EVs represent a compelling frontier for biomarker discovery, with a clear potential to aid in the diagnosis, prognosis, and longitudinal monitoring of complex human diseases including a range of cancers, auto‐immune and neurodegenerative diseases. Salivary EVs provide an alternative approach to costly, invasive, and static biopsies, which are not well‐suited for tracking dynamic disease progression. However, translation from bench to bedside is hindered by a persistent lack of methodological standardization. Furthermore, the absence of consensus on isolation protocols contributes to a landscape of divergent biomarker findings which make cross‐study comparisons challenging.

**FIGURE 7:**
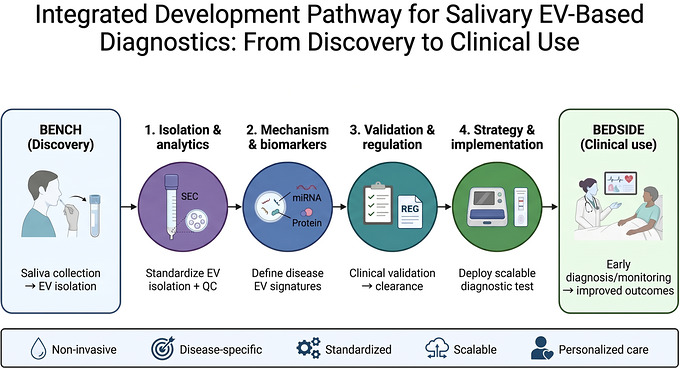
Integrated translational pathway for salivary EV‐based diagnostics, from discovery to clinical use. This schematic presents a bench‐to‐bedside roadmap for translating salivary EV research into clinical utility. At the bench (discovery), saliva is collected as a non‐invasive source, and EVs are isolated. The pathway proceeds through four stages: (1) isolation and analytics, standardising EV isolation and quality control (QC), for example by size‐exclusion chromatography (SEC); (2) mechanism and biomarkers, defining disease‐specific EV signatures from cargo such as miRNA and proteins; (3) validation and regulation, clinical validation leading to regulatory clearance; and (4) strategy and implementation, deployment of scalable diagnostic tests. At the bedside (clinical use), these tests enable early diagnosis and monitoring, supporting improved patient outcomes. The lower banner summarises five cross‐cutting principles underpinning the pathway: non‐invasive sampling, disease specificity, standardised methodology, scalability, and personalised care. Created in BioRender.

To advance the clinical utility of salivary EVs, future research must prioritize rigorous adherence to MISEV2023 (Welsh et al. 2024) guidelines to ensure methodological reproducibility and reporting consistency. Building on this standardized framework, large‐scale, multi‐cohort studies are essential to validate salivary EV‐derived biomarkers and distinguish disease‐specific signatures from physiological variations. Additionally, the field must mechanistically elucidate the influence of the oral microbiome on EV secretion and cargo to minimize confounding factors. Finally, successful translation from bench to beside mandates that studies evaluate the isolation purity alongside economic feasibility, thereby establishing scalable workflows for routine diagnostic application (Figure 7).

## Author Contributions

**Kwanele Xulu**: writing – original draft, visualization, writing – review and editing, validation, conceptualization. **Usri H. Ibrahim**: writing – original draft, writing – review and editing, formal analysis, conceptualization, visualization. **Irene Mackraj**: supervision, resources, writing – review and editing. **Carola Niesler**: supervision, resources, writing – review and editing, visualization, conceptualization, project administration.

## Conflicts of Interest

The authors declare no conflicts of interest

## Data Availability

Data sharing not applicable to this article as no datasets were generated or analysed during the current study.
